# Profiles of Caregiver-Reported Executive Function in Children with Down Syndrome

**DOI:** 10.3390/brainsci12101333

**Published:** 2022-10-01

**Authors:** Kaylyn Van Deusen, Mark A. Prince, Anna J. Esbensen, Jamie O. Edgin, Emily K. Schworer, Angela John Thurman, Lina R. Patel, Lisa A. Daunhauer, Deborah J. Fidler

**Affiliations:** 1 Human Development and Family Studies, Colorado State University, Fort Collins, CO 80523, USA; 2 Department of Psychology, Colorado State University, Fort Collins, CO 80523, USA; 3 Division of Developmental and Behavioral Pediatrics, Cincinnati Children’s Hospital Medical Center, Cincinnati, OH 45229, USA; 4 College of Medicine, University of Cincinnati, Cincinnati, OH 45229, USA; 5 Sonoran University Center for Excellence in Developmental Disabilities, University of Arizona, Tucson, AZ 85721, USA; 6 Waisman Center, University of Wisconsin—Madison, Madison, WI 53705, USA; 7 Department of Psychiatry, MIND Institute, University of California Davis Health, Sacramento, CA 95817, USA; 8 Department of Psychiatry, University of Colorado-Anschutz Medical Campus, Aurora, CO 80045, USA

**Keywords:** executive function, Down syndrome, latent profile analysis

## Abstract

Children with Down syndrome (DS) are at risk for challenges with aspects of executive function (EF). The current study explores whether heterogeneity in EF profiles can be detected within a sample of children with DS. Participants were 69 children with DS, ages 3–10 years (M = 6.23, SD = 1.91). T-scores from a caregiver-report measure of executive function were modeled using latent profile analysis, and auxiliary analyses examined the association between demographic and biomedical factors and probability of profile membership. The two-profile solution was the best fit for the sample, with a profile that involved elevated scores in working memory only (“Working Memory Only” profile; 43% of sample) and a “Multi-Domain” profile that involved elevated scores in planning, inhibition, and working memory (57%). The presence of congenital heart defects was associated with a higher probability of assignment to the Multi-Domain profile. Findings from this study contribute to the characterization of heterogeneous outcomes associated with DS.

## 1. Introduction

Executive functions (EFs) are the cognitive processes required for everyday, goal-directed behavior and are generally thought to encompass working memory, planning, inhibitory control, and cognitive flexibility, among other cognitive constructs [1,2,3]. EF component processes develop throughout early childhood, with precursors of EF hypothesized to emerge during infancy [4,5]. Differences in the development of EF have been characterized in a range of clinical populations [6] and challenges with these cognitive regulatory skills have been identified as a risk factor for neurodevelopmental conditions [7]. More than 20 years ago, it was hypothesized that different clinical conditions could be associated with unique EF “fingerprints” or performance patterns across various component processes that are characteristic of individuals with a given condition [8]. Within this context, there has been significant interest in identifying and characterizing the nature of EF in individuals with broader cognitive delays, such as those with intellectual disability (ID) [9]. Part of this effort has focused on identifying patterns of EF performance in specific neurogenetic conditions associated with ID, including those with Down syndrome (DS) [10,11].

DS is among the most common neurogenetic conditions associated with ID [12]. At the group level, overall EF challenges are observed across nearly all studies of people with DS, regardless of lifespan phase [13,14]. When considering EF component processes, most studies report specific challenges in working memory for participants with DS [10,11,13,15,16,17]. Other studies also identify relative challenges with planning skills during early childhood in DS [15,18,19], but this group-level finding is not consistently reported throughout later childhood and adolescence [11,13]. Inconsistency across empirical studies is also observed when describing outcomes related to inhibitory control, with some studies identifying this EF component as a potential area of challenge for some individuals with DS, but null findings reported in other studies [10,11,15,17]. Finally, the EF component of cognitive flexibility is not a reported area of pronounced challenge during childhood; however, it appears to become more of an evident difficulty in adolescence and early adulthood in this population [11,14,20].

Though much attention has been placed on describing patterns of EF strength and challenge over the past decade, these studies have mainly adopted a nomothetic approach. To date, within-group heterogeneity has been under-examined in the literature for this population. Yet, studies have also reported a distinct subgroup of individuals with DS who demonstrate more pronounced difficulty within the broader category of self-regulation [10,21]. In particular, in comparing children with DS with and without co-occurring attention deficit and hyperactivity disorder (ADHD), key differences are observed in parent ratings of EF, conduct and oppositional behaviors, inattention, irritability, and hyperactivity [21]. These subgroup patterns of children with DS and additional regulation challenges are not well-represented in studies that report group-level findings only. From a translational perspective, taking a group-level or nomothetic approach has important advantages, as it can help educators and interventionists anticipate potential areas of vulnerability and provide early enrichments that may strengthen foundations before areas of strength and challenge become pronounced [22]. However, there is a growing awareness that group-level patterns may obscure the detection of subgroups of individuals who may benefit from more intensive supports, particularly those with co-occurring conditions [23].

In addition to characterizing heterogeneous outcomes within neurogenetic conditions, a related worthwhile pursuit is the identification of factors that account for the presence of heterogeneity along key dimensions. DS is associated with a number of co-occurring biomedical conditions that may influence neuropsychological development. For example, congenital heart defects (CHD) are present in 44.0–55.9% of infants with DS [24,25]. Initial examinations of the association between CHD and developmental outcomes suggested an association with aspects of motor [26], cognitive, and language development [27,28,29,30]. A substantial number of infants with DS are born preterm [31], and this has recently been linked to attention regulation in infancy [32] and ADHD outcomes [33]. Other biomedical dimensions, such as vision and hearing impairments or history of significant illness, have not been examined for their longitudinal link to cognitive outcomes in DS. These are prevalent in this population and warrant examination.

This present study examined whether different profiles of EF performance were observed among 3–10-year-old children with DS. Using mixture modeling, we conducted latent profile analysis (LPA) on caregiver-reported EF domain scores as measured by the Behavior Rating Inventory of Executive Function—Preschool Form (BRIEF-P) [34]. After identifying the best-fitting model, we conducted auxiliary analyses with demographic and biomedical variables to evaluate their association with probability of profile membership. Results from this study have the potential to contribute to the development of more targeted interventions for young children with DS.

## 2. Materials and Methods

### 2.1. Participants

Participants were 69 children with DS and a primary caregiver, drawn from two larger, multi-site studies of cognition in DS. Each project site recruited participants with different chronological age (CA) ranges. One project recruited participants who were 6.00–17.99 years chronologically, and the other project recruited participants ages 2.50–7.99 years. Only children ages 3.00–10.99 years from these two larger projects were included in the present study. For the broader projects, in addition to the specific CA ranges and a DS diagnosis, child/youth participant inclusion criteria were as follows: no more than mild documented hearing loss and no uncorrected vision problems. All parent and caregiver participants completed the assessments and questionnaires in English.

For the present study, additional inclusion criteria comprised having a caregiver-completed BRIEF-P and a mental age (MA) that corresponded with the standardization sample of the BRIEF-P (2.00–5.99 years MA). All participants met this MA criterion. The resulting sample had an MA range of 2.00–5.92 years (M = 2.68, SD = 0.88) and a CA range of 3.04–10.46 years (M = 6.02 years, SD = 1.76). See Table 1 for additional participant demographics.

### 2.2. Procedures

Participants were recruited from two multi-site studies of cognition in children and adolescents with DS. The procedures at each site were approved by professional external Institutional Review Boards (IRBs). Recruitment took place in the Midwest and Mount West regions of the United States through the distribution of flyers in parent advocacy groups, social media, and through local DS clinics. Caregivers completed questionnaires about their child’s adaptive skills, behavior, medical history, and demographic information.

Study data were entered into or directly collected through REDCap (Research Electronic Data Capture), an electronic database for management of research information [35]. REDCap is a secure, web-based application designed to support data capture for research studies, providing (1) an intuitive interface for validated data entry; (2) audit trails for tracking data manipulation and export procedures; (3) automated export procedures for seamless data downloads to common statistical packages; and (4) procedures for importing data from external sources.

### 2.3. Measures

#### 2.3.1. Developmental Status

The *Stanford Binet, Fifth Edition Abbreviated Intelligence Quotient* (SB5-ABIQ) [36] was administered to all child participants. The SB5-ABIQ estimates intelligence through the administration of two routing subtests for individuals 2 to 85 years. One subtest estimates Nonverbal Fluid Reasoning (Object series/Matrices) and a second subtest provides an estimate of Verbal Knowledge (Vocabulary). The routing subtests have high reliability with other IQ scales on the SB5 (+90) [37]. Standard scores and age-equivalent scores are calculated from raw total scores on each subtest [36]. The SB5-ABIQ has a floor of 47 for standard scores and < 2–0 years for age-equivalent scores. Raw scores were transformed into age-equivalent scores according to the procedures in the SB5 manual [36]. The participants scoring at the floor were assigned an age equivalent of 2.00 years for the purposes of deriving an MA for each participant (n = 27). A developmental quotient (DQ) score was calculated for each participant by dividing a child’s MA by their chronological age.

The *Behavior Rating Inventory of Executive Function—Preschool Version* (BRIEF-P) [34] was completed by caregivers to provide an ecological account of their children’s executive function skills in everyday life. This 63-item questionnaire is answered on a Likert-type scale of N (Never), S (Sometimes), and O (Often). The composite score for the measure is a Global Executive Composite. There are three indices: an Inhibitory Self-Control Index, a Flexibility Index, and an Emergent Metacognition Index. The indices can be split into five clinical scales: Inhibitory Control (e.g., “Is impulsive”/“Acts too wild or out of control”) [34], Shifting (e.g., “Is upset by a change in plans or routine”) [34], Emotional Control (e.g., “Mood changes frequently”) [34], Working Memory (e.g., “Needs help from an adult to stay on task”) [34], and Plan/Organize (Planning; e.g., “Has trouble thinking of a different way to solve a problem or complete an activity when stuck”, Organizing; e.g., “When instructed to clean up, puts things away in a disorganized, random way”) [34]. The five clinical subscales of the BRIEF-P were the focus of the present study.

Raw scores for the BRIEF-P were summed from item-level responses into raw subscale scores. For missing responses on individual items, a score of “Sometimes” was entered in the dataset, as per the BRIEF-P Manual guidance [34] (p. 7). Raw scores were then transformed into T-scores using the norming tables of the BRIEF-P [34] (pp. 7, 11). We note that for a subset of participants at one study site, there were three items missing with more systematic missingness due to site-related administration issues (n = 23). The three items with increased missingness were omitted from the calculation of Cronbach’s alpha.

To account for the differential between CA and MA generally observed in DS, T-scores for each BRIEF-P clinical subscale were calculated using a child’s SB5-ABIQ age-equivalent score, rather than their CA [36]. Participants at the floor of the SB5-ABIQ were scored in the youngest (2.00–3.11-year-old) age band on the BRIEF-P. This scoring procedure is consistent with a growing number of studies that have used MA to score proxy-report measures of EF in people with developmental delays [10,16]. Calculating T-scores on the BRIEF-P using MA allows for the comparison of a child with DS to other children at the same MA, not the same CA [10]. This makes it possible to estimate whether EF skills are on par with overall developmental status in a child with DS, or whether more pronounced levels of EF difficulty are observed than would be expected for overall developmental level. A T-score ≥ 65, which is equivalent to 1.5 SDs above the mean, is considered clinically elevated [38]. Cronbach alpha for the total BRIEF-P score in this sample was 0.98, and subscale alpha values ranged from 0.87 to 0.95.

#### 2.3.2. Medical History Questionnaire

Caregivers answered a series of questions about their child’s medical history through a paper form or via the REDCap platform. Medical history questions covered premature birth, CHD, vision or hearing impairment, and other biomedical diagnoses. Data were then transformed into composite variables for the following dimensions by adding the total number of conditions reported: biomedical (e.g., thyroid problems, gastrointestinal concerns, seizures, diabetes, and head injuries) and sensory (e.g., hearing problems, vision problems, and frequent ear infections). Composite scores were created from the individual questions on the medical history questionnaire, as long as there was not more than one missing response for the diagnoses in that group (n = 2 without biological composites, n = 4 composite scores calculated with one question missing).

### 2.4. Analytic Approach

To characterize heterogeneity in parent-reported EF across the five BRIEF-P domains, latent profile analysis (LPA) [39,40,41] was conducted using Mplus version 8 [42]. A series of models (1- through 4- profiles) were estimated and model fit was examined for each model. The best-fitting model was selected based on the following criteria: 1. The Lo–Mendell–Rubin likelihood ratio test of model fit (LMR) [43], which compares current model fit to the model with one fewer profile; 2. The sample size adjusted Bayesian Information Criterion (aBIC; values closer to zero indicating better fit) [44], which is used to compare model fit across nested models [45]; 3. Entropy values (values closer to 1 indicating better fit and 0.80 being a recognized cutoff for adequate classification), which serve as an index of classification quality [46]; and 4. Average latent class probabilities (ALCPs; values closer to 1 indicating good fit and values greater than 0.90 being preferred for indicating good class separation), which represent how well participants fit with their highest probabilistic membership profile. After evaluating each of these fit indices, the substantive interpretation of each model and its parsimony were considered in final model selection.

### 2.5. Auxiliary Testing

After the model selection process, auxiliary analyses were conducted with the best-fitting model to characterize the association between sex, premature birth, CHD, medical history composites (e.g., biomedical and sensory), and CA with profile probability scores. Auxiliary analyses were conducted simultaneously with LPAs to control error and account for probabilistic profile membership. These variables were treated as categorical auxiliary distal outcomes utilizing the BCH and DCAT methods [47,48], and global and pairwise comparisons were estimated using Wald chi-square tests.

## 3. Results

**Figure 1:**
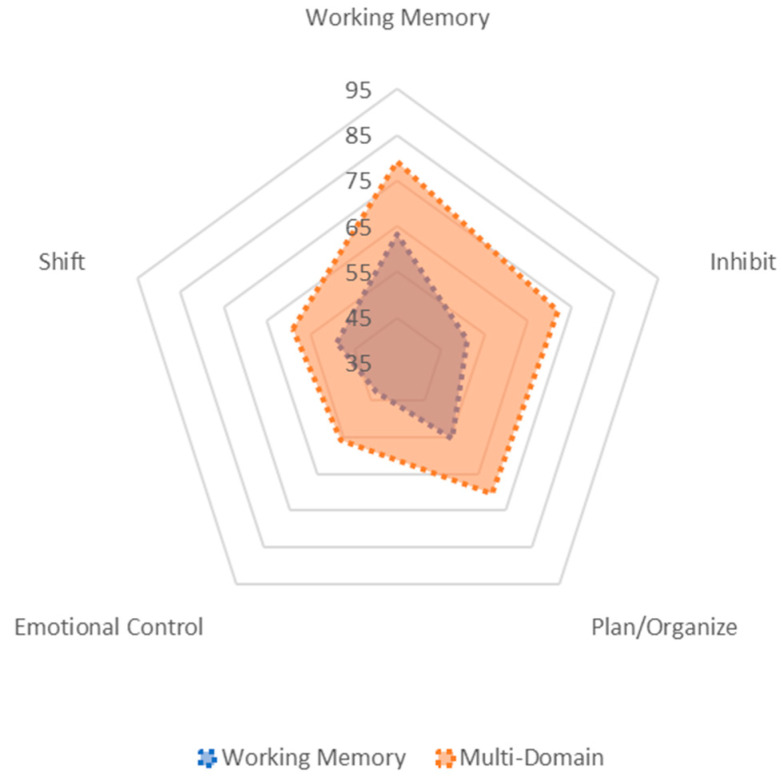
Working Memory and Multi-Domain Profiles radar plot compares the overlap in mean BRIEF-P domain T-scores across the two latent profiles. The scale is set to the range of individual BRIEF-P T-scores (T = 35–104) in the study sample.

The two-profile solution was the best fitting model (see Table 2). The two-profile model had relatively lower aBIC than the 1 profile model and the LMR test confirmed that the two-class solution was a better-fitting model than the 1-class solution. Though the entropy values were marginally higher for the 3- and 4-profile solutions, the LMR tests did not show improvement in terms of model fit and ALCP values were somewhat lower. See Table 3 for means and standard errors for each latent class. In Figure 1, the means for each of the profiles are visualized using a radar plot. This plot compares the estimated mean values in the BRIEF-P domains across the two identified latent profiles. For the two-class solution, one profile provided the best fit for approximately 43% (n = 29) of participants, with scores at 55 or under for all EF domains, with the exception of Working Memory. The mean T-score for Working Memory in this profile was elevated, but below the clinical cut off (Mean = 63.26; SE = 2.65). This profile was termed the “Working Memory Only” profile. The second profile provided the best fit for approximately 57% (n = 40) of the participants in the sample, with elevated mean scores for Working Memory, Inhibition, and Planning/Organizing (all Means 71–79), and T-scores under 60 for Shifting and Emotion Control. This profile was termed the “Multi-Domain” profile.

### Auxiliary Testing Results

Auxiliary tests were conducted with the dimensions of sex, CA, MA, DQ, CHD, premature birth, and the biomedical and sensory impairments composite variables; results are presented in Table 3. Only one dimension was associated with probability of profile membership: CHD. The Multi-Domain and the Working Memory profiles differed significantly on the presence/absence of CHD, with participants with CHD more likely to have the Multi-Domain profile as their best fitting profile, X^2^ (1) = 3.93, *p* < 0.05. It is noted that the Working Memory profile was associated with significantly older MAs, X^2^ (1) = 4.30, *p* < 0.04. However, when DQ was evaluated, no statistically significant associations were observed with probability of profile membership. This suggests that the MA associations were not a function of degree of developmental delay; rather, they were likely an artifact of the characteristics of this sample. No other statistically significant associations were observed in auxiliary analyses.

## 4. Discussion

This study examined whether heterogeneity was observed among young children with DS, ranging from 3.00 to 10.99 years old, on a parent-reported ecological measure of EF. Using LPA, the best model fit for this sample was a two-profile solution, yielding one profile that involved mainly challenges with working memory (“Working Memory Only”), and another profile that involved challenges with inhibition and planning/organizing, along with elevated challenges with working memory (“Multi-Domain”). Figure 1 compares these profile differences by visualizing the estimated means for each domain. Auxiliary analyses demonstrated an association between the presence of CHD and probability of profile membership such that children with DS who had co-occurring CHD were more likely to be assigned to the Multi-Domain profile.

These LPA findings are of note because they expand our current understanding of the various EF presentations generally observed in children with DS. First, our findings replicate the well-characterized working memory challenges generally observed in people with DS across proxy-report and direct assessment study designs. Both profiles in the best-fitting model demonstrated elevated levels of difficulty in this area, with average working memory T-scores more than one standard deviation above the MA-mean in the Working Memory Only profile (profile M = 63.26) and more than two standard deviations above the mean in the Multi-Domain profile (profile M = 79.11). Thus, working memory challenges that are more pronounced than expected for developmental level appear to be relatively homogeneous among people with DS, differing mainly in terms of degree.

Findings from this study also clarify that working memory appears to be the primary, and perhaps the only, EF area of challenge relative to MA-expectations for only some participants in our sample. The second profile observed in this study, which was a fit for approximately 57% percent of participants, involved difficulties with working memory, and accompanying challenges with inhibition and planning/organizing. For participants likely to be assigned to this profile, mean scores for Working Memory were above 70, and mean Plan/Organize and Inhibit scores were above 70 as well. These findings shed light on somewhat contradictory results in the EF literature in DS. Though numerous studies have identified relative challenges in all EFs relative to CA norms [11,14], and pronounced working memory challenges in the majority of studies, inhibition and planning have either been characterized as “intermediate skills” [11] or studies have shown contradictory findings, with some studies identifying these dimensions as areas of challenge and others reporting no apparent relative challenges [10,49,50,51]. Because previous studies involved mainly group-level study designs, it may be the case that the “intermediate” challenges represent the average across the two profiles identified in this study, one with elevated inhibition and planning skills, and one without. Studies with contradictory findings may have included differing proportions of participants from one profile or the other. Future examinations of EF-related findings in DS should account for these profiles in their study designs and include a careful account of medical comorbidities to provide a more comprehensive account of EF presentations in DS, which can ultimately inform more tailored and personalized educational plans.

*Biomedical factors*. A noteworthy finding reported in this study is the association between CHD and the probability of profile membership. Participants with DS in this study who had a CHD were more likely to be assigned to the Multi-Domain profile. These findings warrant replication, as it suggests that cognitive and developmental consequences for children with DS born with CHD are evident throughout childhood. Earlier studies have reported that CHD has short-term implications for overall cognitive and communicative development; however, subsequent longitudinal work has not consistently demonstrated these effects [27,28]. In the present study, a longer-term outcome associated with cognitive regulation was observed, and these findings are aligned with the larger literature on CHD and EF in the general pediatric population [52,53]. Children born with various types of CHD are known to demonstrate a range of EF challenges, with the severity of the CHD diagnosis associated with the degree of EF challenge [52]. The well-replicated finding of an association between CHD and EF challenge in the general pediatric population provides additional justification for future examination of vulnerable pathways in children with DS, and the long-term consequences of additional biomedical risk factors.

These CHD-related findings stand in contrast to the null findings observed in the relationship between EF profile and other potentially relevant domains, such as premature birth, sex, CA, and composite biomedical and sensory impairment variables. Though previous research on infants with DS has demonstrated an association between cognitive dysregulation and premature birth [32,33], no meaningful association of this nature was observed in the present study. This may be attributed to the difference between direct observation measures versus proxy report data, or it may be the case that numerous other factors subsequent to the perinatal period exert a greater influence on the development of EF, such that these effects are no longer detectable between the ages of 3.00–10.99 years. Similarly, no effects for sex were observed in this study, which replicates previous findings using the BRIEF with children with ID more broadly [54]. CA-related differences were not statistically significant, though it is notable that the Working Memory Only profile was associated with a CA that was approximately 1 year older than the Multi-Domain profile. In addition, though a significant association was observed between MA and probability of profile membership, there was no such association when DQ was examined, which controls for CA in relation to MA. This suggests that overall developmental status or degree of delay was not a significant factor associated with the profile outcomes observed.

### 4.1. Implications

Findings from this study contribute both to the growing literature on EF in DS, and to the growing account of heterogeneity in outcomes among those with DS. DS is generally associated with a pattern of phenotypic strength and challenge in various developmental domains [55]. For example, the phenotypic profile associated with DS tends to include relatively stronger visual than verbal processing, stronger receptive than expressive language, and challenges in the development of gross and fine motor skills. However, a great deal of heterogeneity across these outcomes is also observed [56]. For example, while many individuals with DS develop MA-appropriate levels of early social communication [57], a subgroup of children with DS have co-occurring autism spectrum disorder diagnoses and demonstrate more pronounced difficulty with the development of social communication skills [58]. This within-syndrome variability is observed across nearly every domain of functioning studied, including overall cognitive status [59], motor development [60], language acquisition [61], and various aspects of social relatedness [62].

Group-level findings have unquestionably advanced our appreciation of phenotypic predispositions associated with specific neurogenetic conditions. Addressing within-syndrome heterogeneity through the use of more advanced analytic techniques, such as mixture modeling, can further refine the anticipatory stance afforded to clinical researchers and interventionists in neurogenetic syndrome research [23]. Identifying early profiles that confer additional risk for children with a specific condition, such as the Multi-Domain profile observed in the DS sample in this study, makes it possible to generate recommendations for clinicians and researchers about which children may benefit from early enrichments before pronounced profiles of challenges are evident in later childhood or adolescence. Studies that address heterogeneity can potentially refine our intervention techniques and tailor educational planning.

Given the presence of a subgroup of participants whose EF profiles involve greater degrees of difficulties with regulation, future work should seek to examine the link between these profiles and the risk for a diagnosis of ADHD. As Esbensen et al. [21] reported, children with co-occurring DS and ADHD demonstrate a range of behavioral and cognitive regulatory differences compared to those with DS only. An early childhood presentation of greater challenges than predicted by MA in working memory, as well as inhibition and planning, may potentially become a valuable indicator of elevated risk for a co-occurring diagnosis, which may facilitate more targeted and anticipatory intervention.

### 4.2. Limitations

This study advances our understanding of EF in DS, but its findings must be interpreted in the context of several limitations. First, the BRIEF-P is a proxy report measure that relies on the evaluation of a family member, educator, or clinician for reports of everyday EF performance. Although some have argued that proxy-report measures such as the BRIEF-P establish ecological validity in the area EF measurement, reporter bias may also influence the study’s findings. Furthermore, we note that proxy-report measures of EF have not consistently shown convergence with laboratory-based assessments within individuals with DS [15]. In light of this issue, we note that these findings may not align with other studies of laboratory-based measures of EF that report no meaningful group differences in cognitive tasks among preschoolers with DS [63].

In addition, as noted above, the calculation of T-scores was conducted using a child’s MA as derived from the SB-5 ABIQ, not CA. This is a departure from the traditional approach to standard score calculation, and from the procedures in the BRIEF-P manual [34]. Virtually all children with DS demonstrate significant delays relative to CA expectations. There is a critical need to elucidate patterns of strength or challenge experienced by children across EF domains relative to cognitive-level expectations. There are no available tools that have been validated for use in young children with DS that allow investigators to consider developmental comparisons across EF domains. Thus, in the present study and several other investigations of EF development in children with DS [10,16], the present approach to calculating T-scores has been used as a first step in considering EF performance relative to a child’s overall developmental status, rather than the comparison with CA. Nevertheless, these findings should be interpreted with this methodological deviation in mind. Age-equivalent scores represent the median level of performance of children included in the norming sample at that designated age and are not ratio or interval scales of measurement [64]. Thus, they are an imperfect estimate of developmental level. Additional research aggregating cohorts to create reference samples of BRIEF-P data for children with DS across norming ages and/or validating EF measures across multiple subdomains in young children with DS is needed.

Another important issue to consider relates to the use of the SB-5 ABIQ to estimate developmental levels in the present study. Although researchers studying children with DS have begun to converge on the use of the SB-5 with recommendations regarding the use of deviation scores [65,66], the SB-5 nonetheless includes both verbal and nonverbal components that contribute to its brief IQ estimates. For children with DS, who are likely to show language scores that lag scores for nonverbal cognitive ability [67], this overall IQ score estimates a developmental level that will fall between domain performance scores. Additional investigations considering EF development in children with DS relative to both nonverbal and language ability will help clarify the intricate associations across these developmental domains. Furthermore, a subset of children in the present study had age-equivalent scores at the floor (below two years of age), which is a consequence of the use of normed IQ measure with young children with ID.

An additional limitation of this study is that participants were recruited through community samples. Therefore, caution must be taken in the interpretation of the percentages representing each profile presented in the results. In this sample, a larger percentage of participants had the Multi-Domain profile as their likely profile when compared to the Working Memory Only profile. This could be an accurate reflection of the larger population of participants with DS, or it may be the case that families of children with greater degrees of cognitive regulation difficulties were more likely to enroll in research studies on cognition in DS. Future studies should examine whether the two-profile model is the best fit for other samples of participants with DS. If this finding is replicated, investigators should also consider whether the distribution of likeliest profile assignment was similar to that which we observed in the present study.

It is also important to consider the CA range in this study. Because the focus of the study was on childhood EF, only participants younger than 10.99 years were included from the larger studies on cognition in children with DS. As a result, the observed profiles should be interpreted only as reflective of this particular age range of participants. One implication of this methodological choice is that elevated challenges with shifting were not observed in either profile. Previous studies have reported that shifting challenges become more pronounced in adolescents and adults with DS, and are less evident in younger children [11,13]. Caution should be taken when interpreting the present study findings, as cognitive flexibility may be a potential challenge for people with DS during later phases of the lifespan, though challenges were not observed in the present sample of young children. Relatedly, another consequence of the present cross-sectional study design is the lack of in-depth, longitudinal information regarding the association observed between CHD and EF profiles in this sample. Numerous factors were unexplored in this study, including a history of surgical correction and the timing of surgery, the type of CHD, and longitudinal data regarding trajectories associated with these factors. Future studies should examine this connection in greater depth, with a more intensive longitudinal study design.

This study also included a smaller sample size than is generally observed in studies that use an LPA approach, though the sample is relatively larger than samples included in many studies of young children with DS. It is noted, however, that simulation studies (e.g., Lubke & Neale [68] have shown that model selection depends on class separation. The present sample size (n = 68) was adequate to detect the large class separation in our data, as indicated by the high classification quality. Future studies should be conducted to identify whether similar findings are observed in larger samples. This study is also limited in that participants were primarily White and all families had caregivers who reported at least some post-secondary educational training. Representativeness in DS research is a critical issue both ethically and to ensure the most accurate findings that are applicable to as many individuals with DS and their families as possible. Future efforts should be made to ensure greater representation in studies on EF in children with DS. In addition, though caregivers were asked to provide information about child biomedical conditions, some conditions may have not yet been diagnosed, especially for the youngest participants. As a result, some biomedical associations may not have been detectable.

Though these limitations should be taken into consideration in the interpretation of the findings of this study, the results presented here are nonetheless informative and contribute to our growing understanding of the profiles of outcomes that can be observed among children with DS. Examinations of heterogeneity within DS and other neurogenetic conditions are a necessary next phase in the study of phenotypic profiles of strengths and challenges associated with specific neurogenetic conditions. By understanding more subtle performance profiles, researchers and clinicians are then able to ask questions about sources of this heterogeneity, identify modifiable factors that can be targeted in personalized ways, and this newfound information can be used to motivate more informed and effective educational and intervention approaches.

## Figures and Tables

**Table 1:** Demographic Information.

Child Characteristics	Mean (SD) or % (n)
% Male	44.9 (31)
Child Chronological Age (years)	6.23 (1.91)
Child Mental Age (years)	2.68 (0.89)
Race (n = 1 missing)	
Asian-American	5.90 (4)
Black/African American	2.90 (2)
White	88.2 (60)
Other	2.90 (2)
Ethnicity (n = 3 missing)	
Hispanic	13.60 (9)
Not Hispanic	86.40 (57)
DS Type (n = 1 missing)	
Trisomy 21	91.2 (62)
Mosaicism	2.9 (2)
Translocation	2.9 (2)
Not Sure	2.9 (2)
Premature Birth (% yes; n = 2 missing)	23.9 (16)
Congenital Heart Defects (% yes)	63.8 (44)
**Caregiver Characteristics**	
Primary Caregiver Age (n = 1 missing)	41.75 (6.43)
% Primary Caregiver Education at least 1 year of college/tech training	98.6 (68)

**Table 2:** LPA Models: comparative fit and classification quality.

	1-Class	2-Class	3-Class	4-Class
aBIC	2683.167	**2582.681**	2539.631	2511.826
Entropy	−	**0.84**	0.898	0.889
ALC-Probabilities	−	**0.96 to 0.96**	0.93 to 0.96	0.91 to 0.99
LMR Test (value, *p*-value)	−	**102.941, 0.021**	47.681, 0.413	33.013, 0.348
	**Number in Most Likely Class Count (Proportion)**			
Class 1	69 (100%)	**29 (43%)**	18 (26%)	5 (7%)
Class 2		**40 (57%)**	8 (12%)	7 (10%)
Class 3			43 (62%)	30 (43%)
Class 4				27 (39%)

aBIC = sample size adjusted Bayesian Information Criterion; ALC-Probabilities = Average Latent Class Probabilities for Most Likely Latent Class Membership; LMR = Lo-Mendell-Rubin Likelihood Ratio Test. Bold indicates the selected best fitting model.

**Table 3:** Best fitting model means by class and auxiliary variable testing summary.

	BRIEF-P Domain Models	
	Working Memory N = 29 M (SE)	Multi-Domain N = 40 M (SE)
Working Memory	63.26 (2.66)	79.11 (2.08)
Plan/Organize	55.41 (2.23)	70.34 (1.88)
Shift	49.01 (1.94)	59.09 (1.92)
Inhibit	51.11 (2.26)	71.75 (2.03)
Emotional Control	43.00 (1.70)	55.91 (1.62)
	**Auxiliary Testing** **Mean or Proportion in Each Class**	
Chronological Age (Mean in yrs)	6.77	5.83
Sex (proportion)		
Male	0.56	0.36
Female	0.44	0.64
Sensory Composite (Mean)	0.85	1.14
Biomedical Composite (Mean)	0.92	1.14
DS Type (proportion)		
Trisomy 21	1.00	0.84
Mosaicism	0	0.05
Translocation	0	0.05
Not Sure	0	0.05
Premature Birth (proportion)		
No	0.80	0.71
Yes	0.20	0.26
Do not know	0	0.03
* Congenital Heart Defect (proportion)		
No	0.52	0.24
Yes	0.48	0.76
* Mental Age (MA) Developmental Quotient (MA/CA)	35.66 0.46	29.64 0.44

BRIEF-P = Behavior Rating Inventory of Executive Function-Preschool; M = mean; SE = standard error; n = approximate sample size based on participants’ most likely class membership. * = significant difference between Working Memory versus Multi-Domain Profile.

## Data Availability

The data that support the findings of this study are available on request from the corresponding author (D.F.).
